# Laser Therapy: An Alternative Approach for the Management of Halitosis

**DOI:** 10.7759/cureus.65911

**Published:** 2024-07-31

**Authors:** Vaishnavi R Waikar, Neha Pankey, Vidya Lohe

**Affiliations:** 1 Pedodontics and Preventive Dentistry, Sharad Pawar Dental College and Hospital, Datta Meghe Institute of Higher Education and Research, Wardha, IND; 2 Oral Medicine and Radiology, Sharad Pawar Dental College and Hospital, Datta Meghe Institute of Higher Education and Research, Wardha, IND

**Keywords:** microorganisms, preventions, oral hygiene practice, laser therapy, halitosis

## Abstract

Halitosis is the result of combining the Greek word "osis" (pathological alteration) with the Latin phrase "halitus" (breath). No matter what the cause, halitosis can be described as the presence of a foul odour. Effective treatment of halitosis may be facilitated by knowledge of the illnesses and factors that contribute to the condition as well as its symptoms. To properly diagnose and treat patients, healthcare professionals, general practitioners, and dentists must comprehend the cause of the ailment and the best course of treatment. A prevalent issue affecting a large proportion of the global population is halitosis. In 90% of cases, the onset of this illness is oral; however, in 10% of cases, it is systemic. The primary source of the unpleasant smell is the volatile sulphur compounds that Gram-negative bacteria create. The majority of halitosis sufferers find their disease humiliating which makes it difficult for them to go about their daily lives and social lives. A thorough examination was carried out. An evaluation of the tongue was done. The halitosis was treated in afflicted areas using the "Epic Biolase Laser". It is the most robust dental laser and has a low power output which uses a solid-state diode to produce invisible infrared radiation. This device operates aseptically and offers more convenience due to its wireless design, which is powered by a Bluetooth foot pedal. This is used to lower the number of bacteria, and then periodic maintenance is performed to keep them under control. Benefits and outcomes were accomplished. Laser therapy destroys the bacteria that produce volatile compounds and efficiently treats foul breath for a longer period of time. But with a combination of conventional techniques, the results we obtain are even better.

## Introduction

The American Dental Association describes the Latin term "halitus" (breath) and the Greek word "osis" (pathological alteration) as combined, halitosis emerged. Halitosis is characterized as the presence of an unpleasant odour in exhaled air, regardless of the reason. Most halitosis patients are embarrassed by their illness, which interferes with their social life and everyday activities [1]. Furthermore, poor breath may signal an underlying ailment. Understanding the conditions and causes that contribute to halitosis and associated symptoms could aid in the effective management of this ailment. Healthcare practitioners, particularly primary care physicians and dental experts, must understand the disease's genesis and suitable therapy to identify and treat patients correctly.

Halitosis is classified as genuine halitosis, pseudo-halitosis, and halitophobia. "Genuine halitosis" is an odour that is not acceptable among people [2]. "Pseudo-halitosis" is when a patient complains about having awful breath, but others cannot use scientific testing to identify it clinically [3]. Despite the fact that the issue has been fully resolved and there is no social or clinical evidence to back up their concerns, patients still think they have terrible breath. The term "Halitophobia" refers to this disorder [4].

Volatile sulphur compounds (VSCs) are primarily responsible for bad breath. They are created when anaerobic Gram-negative bacteria, such as "Eubacterium", "*Bacteroides forsythus*", "*Tannerella forsythia*", "*Porphyromonas gingivalis*", "*Treponema denticola*", and "*Fusobacterium nucleatum*", act on substrates containing sulphur in the oral cavity [5-7]. These bacteria's metabolism produces "hydrogen sulphide" (H2S), "methanethiol" (CH3SH), and "dimethyl sulphide" (CH3SCH3)-containing VSCs. These substance concentrations are used to detect halitosis [8-11]. However, the prevalence of halitosis may be underestimated because some people may not even be aware of their poor breathing circumstances [12]. When these individuals start interacting with others, the issue intensifies and leads to social isolation within the larger group. It has been found to be disgusting and primitive, and patients even feel ashamed of it [13,14]. Halitosis may also, to some extent, have an impact on community members' productivity at work and the related healthcare system.

## Case presentation

**Figure 1:**
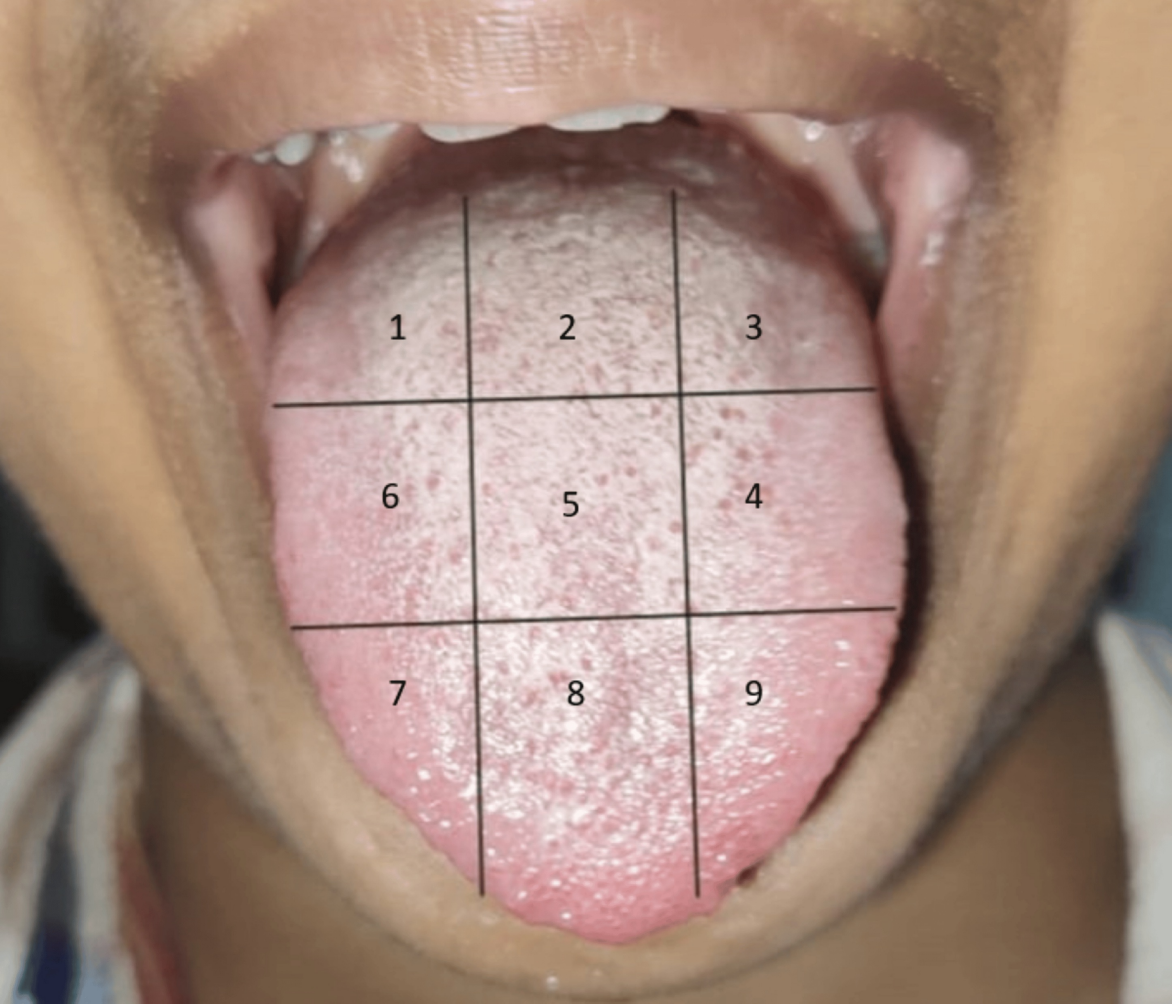
Preoperative image of the tongue showing sections indicates a light or heavy white coating on the tongue.

A 16-year-old patient presented with a chief complaint of bad breath for one year. The patient was alright one year ago, but then she started noticing bad breath and a white coating on the tongue, which was scrapable. Medical history was not significant. The patient gave a past dental history of relapse for three years because of which there were carious teeth and poor oral hygiene. In the morning, it worsens because of improper brushing technique and consumption of certain foods like garlic, onion, and spices which leave the odour throughout the day. An intraoral examination was carried out. All teeth were present in the oral cavity. Occlusal caries were present on the tooth numbers 16 and 17. The tongue was coated with a white layer as shown in the preoperative image of the tongue (Figure 1). There were no grooves and fissures present. Retention of food and plaque was seen on the anterior. The indices were made on the tongue, and it is separated on the tongue into six or three sections based on the presence of tongue coating in each region. Now, each region could be categorised as light or heavy [15]. In the corners of the tongue from right to left in cross-checkered method, i.e., first, third, fourth, sixth, seventh, eighth and ninth region, we can see there is a light layer on the tongue. As on the second and fifth region, there is a heavy white coat present which is the centre of the tongue.

Evaluation of the tongue is done in this next stage, where it is to use their sense of smell to conduct an "organoleptic examination" or assessment. In order to do this, the patient must follow a set of instructions before being assessed which are to stay away from spicy foods, onions, and garlic over the past one or two days, do not use scented cosmetics, food, coffee, or oral hygiene products in the last eight hours, and should not consume any water for the last three hours [16]. The "Rosenberg scale" is one of the organoleptic scales which was used to record the intensity of an odour. In order to assess the effectiveness of anti-odour agents, the 0-5 organoleptic scale is frequently used in breath research and trials, where 0 represents no odour to 5 being the overpowering odour.

**Figure 2:**
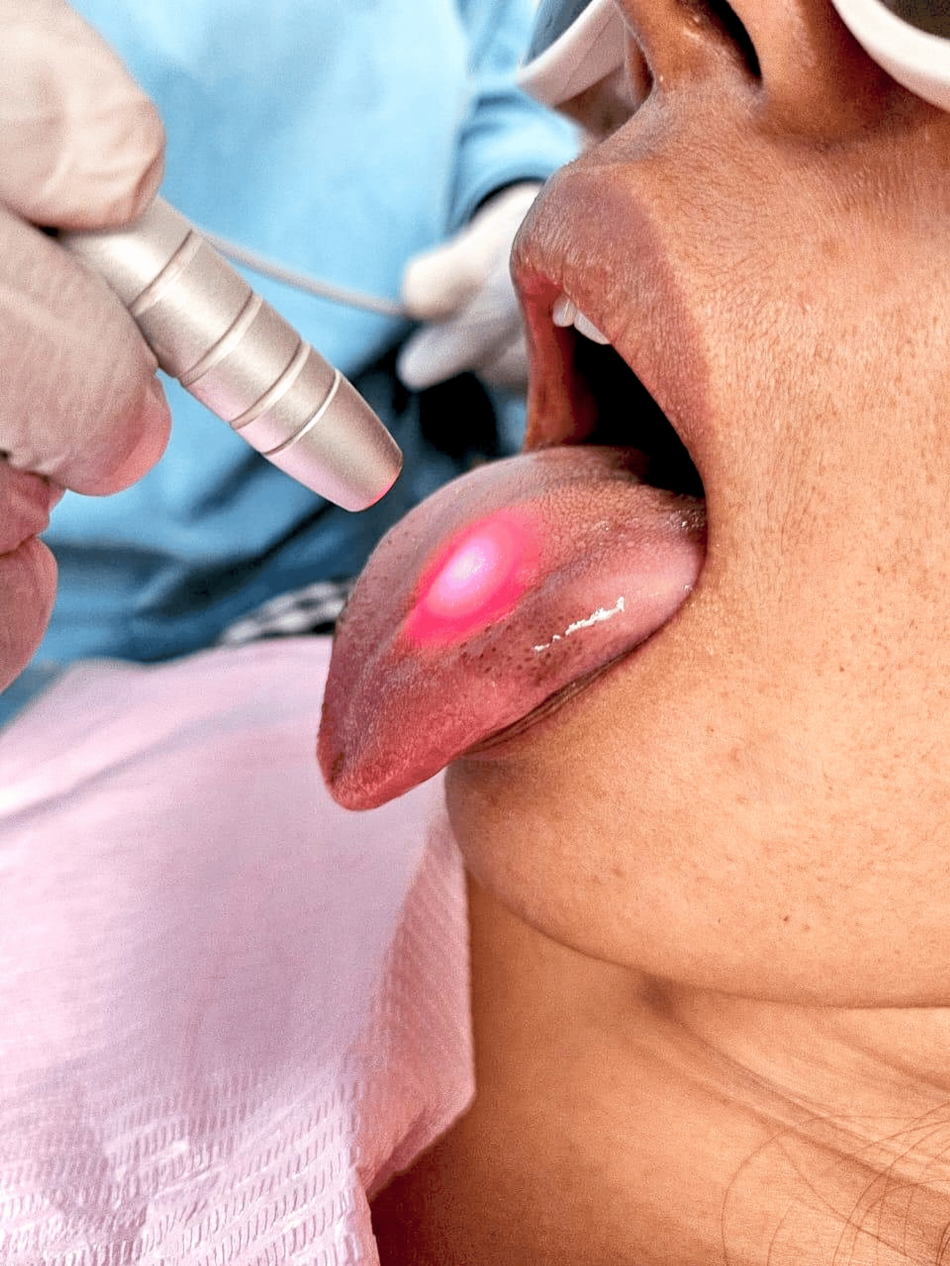
Photograph showing Epic Biolase Laser.

The "Epic Biolase Laser" is a most robust dental lasers. It is a semiconductor medium which generates invisible infrared radiation by using a solid-state diode. The manufacturer named it "BIOLASE" as it is originated from the USA. Various tips are designed to be used in different types of surgical procedures. This device operates aseptically and offers more convenience due to its wireless design, which is powered by a Bluetooth foot pedal. A distinctive feature improves patient comfort by preventing overheating of surrounding tissues and reducing the risk of tissue charring. As shown in Figure 2, it is used on affected areas of the tongue to treat halitosis. As it is a low-power energy laser, laser emission is 980 nm followed with the adjuvant of 3% hydrogen peroxide irrigation in a continuous and non-contact mode with a beamspot area of 0.094 cm and irradiation region of 0.254 cm^2^ per point. When laser was used, irradiation parameters were 10 μsec/pulse duration, 10 kHz, pick power of 10 W, and average power of 1 W. They are evenly distributed uniformly over the target area of tongue. This is used to reduce bacteria, followed by periodic maintenance afterwards to control the bacteria. The duration of the treatment lasted for four to five sessions. Four weeks after treatment, patients were re-examined, and at that point, if additional laser treatment was necessary, it was decided. There were no adverse impacts or complications. Carious teeth were restored, and "Ultrasonic scaling" was done to remove the debris and plaque from the teeth.

In combination with this, there are some conventional techniques which can be used to kill the bacteria that include "mouthwash", "chewing gum", and " lozenges" that usually contain alcohol, zinc, chlorhexidine, and essential oils. Using a tongue scraper is also advised. These techniques, however, are only effective for brief periods of time. There is a search on for alternative safe ways to eliminate or lessen the odours. In addition to cleaning all tooth surfaces and removing plaque, a good dental brushing routine also pays attention to cleaning under the gingival line. Although many modern toothbrushes are made to help with this, most patients are not persistent or skilled enough to perform the essential oral exercises. A proper modified bass brushing technique must be used. An easy and efficient solution is electric brushes [17]. Without consistent daily flossing, halitosis will never improve. Additionally, it needs to be done efficiently, reaching below the gingival line to get rid of any hidden plaque. The tongue is the largest reservoir of bacteria that produce vitamin C, although the second largest spaces are the gingival pockets between teeth. Make sure to utilize any additional interdental cleaning tools that have been prescribed to you on a daily basis. Just scrubbing your tongue is insufficient. It is necessary for you to use an appropriate tongue-cleaning tool. You may find a lot of these cleaners at pharmacies and dental offices. Instead of brushing, the tongue cleaners are used to remove plaque from the tongue. Additionally, they exfoliate the dead skin cells from the skin's outermost layers. This tissue produces an excellent environment for the bacteria that produce volatile sulphur compounds (VSCs), which in turn adds to sour breath. Cleaning the tongue as far back as possible is also crucial. This is accomplished by scraping with the tongue extended as far as feasible. One gram of alum and a pinch of rock salt dissolved in a glass of warm water which should be use to gargle your mouth should be used to treat bad breath and it can strengthen the gums too [18].

**Figure 3:**
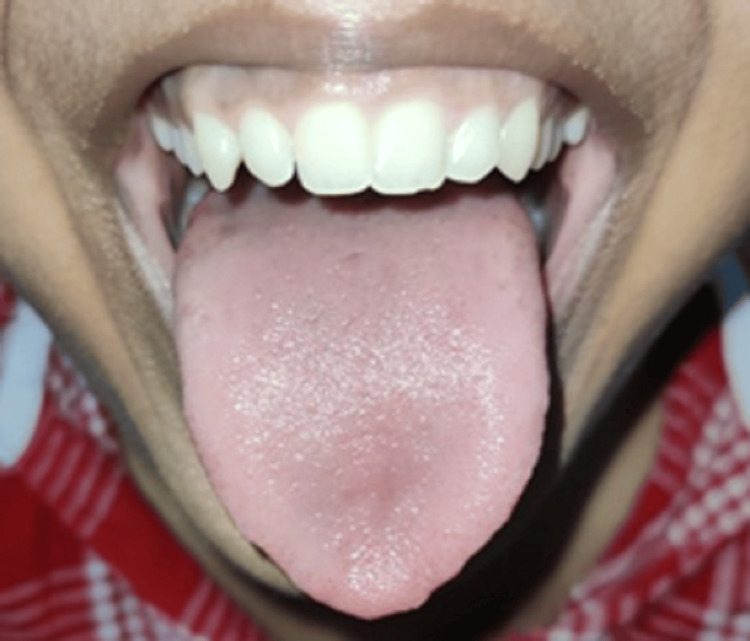
Postoperative image of tongue.

Following the forementioned cleanliness precautions, using an effective antibacterial mouthwash helps eliminate any remaining VSC-producing germs and inhibits their growth. For this, Listerine and its generic counterparts are suitable. Peridex, a prescription mouthwash, is even more effective in circumstances where resistance is higher. If at all feasible, the mouthwash should be thoroughly swished between the teeth, over the tongue, and in the throat for a minute. Additionally, some mouthwashes with more recent formulas say they can break down volatile synthetic compounds (VSCs). Follow-ups were taken every six weeks for one year. The changes were seen in the oral cavity. The tongue was appeared to be (normal) pink in colour, and the oral hygiene was improved as we can see the postoperative image of tongue (Figure 3). It also reduces the chance of gingival disease and encourages healthy gums.

## Discussion

The efficiency of the Epic Biolase Laser in the current study for the treatment of adolescents was evaluated. Bad odours were eradicated when a laser was applied to the dorsum of the tongue, which terminated the concentration of VSCs. A similar study was carried out in which the author stated many combinations of light and photosensitizers have been used to thoroughly examine the efficacy of photodynamic therapy (PDT) on microorganisms. The types and concentration of photosensitizers, the fluence and fluence rate of the light, and the genera of microorganisms all affect how much photodamage occurs. The majority of examined microorganisms are sensitive to PDT, such as *Salmonella typhimurium* and *Escherichia coli*, while *Candida albicans* need a greater dosage [19].

A study was conducted in which it was shown that using a tongue scraper in conjunction with mouthwash can reduce the number of bacteria on the coated tongue, albeit slightly and over an extended period of time [20]. In this study, uneven properties of the tongue’s surface are linked to this restricted decrease in bacteria, which emphasizes the necessity of daily oral hygiene management to sustain a low level of bacterial multiplication [21]. When it came to lowering oral VSC levels, the tongue cleaner-a-brush and scraper combo performed marginally better than the tongue scraper and a standard toothbrush. However, the therapeutic effectiveness of the treatment in lessening oral malodour is still debatable due to its short duration [22]. It provides long-term efficacy on intraoral halitosis both with and without adjunct tongue scraping with the effects of a mouth rinse with zinc acetate (0.3%) and chlorhexidine diacetate (0.025%) [23]. Although it decreased organoleptic scores and the tongue coating index, using a tongue scraper did not enhance the advantages of the active mouth rinse [24]. Therefore, the current study suggests that the Epic Biolase laser can treat halitosis with promising results. Still, it is possible that the best outcomes might come from combining the two approaches.

The benefits and results we achieve after the treatment are long-lasting fresh breath (by addressing the underlying reasons for foul breath, our treatments yield effects that last). Laser therapy lowers the risk of gum disease and promotes gum health in addition to treating halitosis. In this way, oral health is improved. It is a comfortable non-invasive and safe procedure. You will notice a difference in your social interactions and sense of self when you have new breaths, which helps to boost your self-esteem.

## Conclusions

Applying laser therapy to the dorsum of the tongue showed promising outcomes, and it might be recommended as a rapid, efficient, conservative, noninvasive treatment for halitosis in adolescents. Four weeks after treatment, patients were re-examined and for every six weeks for a year, follow-ups were conducted. Compared to conventional techniques of treating this condition, laser therapy successfully eradicates bacteria that create volatile chemicals and will effectively wipe off bad breath for a prolonged duration of time. A problem that is underappreciated by people worldwide is halitosis. It significantly affects social disengagement and quality of life. This social issue needs to be resolved, and remedies that require the least amount of intrusion are needed.
